# Optic Disc Neovascularization as the Only Sign of Ocular Ischemic Syndrome: A Case Report

**DOI:** 10.7759/cureus.29972

**Published:** 2022-10-06

**Authors:** Youstina Metry, Sobha Joseph

**Affiliations:** 1 Department of Undergraduate Medical Education, Heartlands Hospital, Birmingham, GBR; 2 Department of Ophthalmology, Heartlands Hospital, Birmingham, GBR

**Keywords:** ocular ischemic syndrome, optic disc neovascularization, asymptomatic, hyper-fluorescence, carotid endarterectomy

## Abstract

Ocular ischemic syndrome (OIS) features ocular changes occurring secondary to carotid artery occlusive disease (CAOD). We present a unique case of a 64-year-old patient who was referred to the retina clinic due to an incidental isolated finding of left optic disc neovascularization (NVD). The patient was asymptomatic with visual acuity of 6/6 unaided and with normal intraocular pressure bilaterally. Fundus fluorescein angiogram showed hyper-fluorescence on the left optic disc with no areas of capillary dropout. Carotid Doppler and CT angiogram showed significant stenosis within the left proximal internal carotid artery (ICA) and poor visualization of flow in the distal ICA. The patient was urgently referred to the vascular team, and within four months of establishing an OIS diagnosis, she had carotid endarterectomy. As a result, NVD did not show any further progression. This case highlights the importance of community retinal screening by optometrists; that OIS can be asymptomatic, the need to consider CAOD in cases of NVD; and the importance of a multidisciplinary approach.

## Introduction

Ocular ischemic syndrome (OIS) is a rare condition, but it can impose a serious threat to vision. Its seriousness can be overlooked since it can be present with no or unspecific ocular symptoms [1]. It has been estimated that clinical signs of OIS can be incidental and asymptomatic findings in 20% of cases [2]. Symptoms can range from ocular pain to complete visual loss. The mean age for its occurrence is of 65 years with no racial predilection [1,3].

The underlying cause of OIS is severe carotid artery occlusive disease, which causes ocular changes secondary to ocular hypoperfusion. Ocular perfusion abnormalities are noted when stenosis in the common carotid or the internal carotid artery (ICA) reaches about 70%. On presentation with ocular perfusion abnormalities, half of the cases have 100% occlusion of the artery and about 10% have severe bilateral stenosis [3]. OIS, therefore, can unearth this serious underlying pathology that has possible life-threatening consequences.

The ocular changes seen in patients with OIS vary and not all are well known. There is limited information in the existing literature on the presence of optic disc neovascularization (NVD) as the only posterior segment change in ocular ischemic syndrome [3,4]. This report emphasizes the value of this abnormality. Liaison with appropriate specialists was important for this patient to prevent further ocular and life-threatening complications from carotid artery stenosis.

In this case report, we will review the essential aspects of the epidemiology, pathophysiology, and management of OIS.

## Case presentation

A 64-year-old woman was referred by the optometrist to the ophthalmology department due to concerns about abnormal vessels found in the left optic disc. She was known to have hypothyroidism, asthma, and hyperlipidemia. She was on statins and aspirin. She did not have any visual complaints about the presentation.

**Figure 1:**
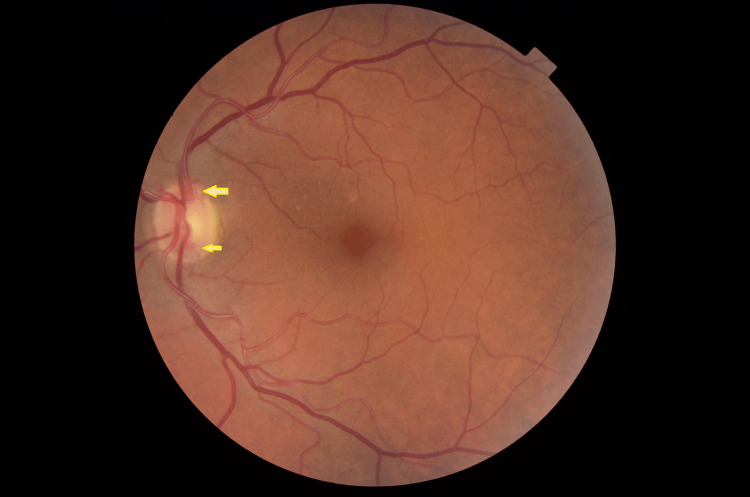
Fundus image of the left eye. Left optic disc neovascularisation (arrows).

On examination, she had visual acuity of 6/6 unaided in both eyes, with intraocular pressure (IOP) within the normal range in both eyes. IOP measurements on presentation were 17 and 18 mmHg in the left and right eye, respectively. On fundoscopy, she had optic disc neovascularization (Figure 1) and occluded blood vessels in the temporal retina in the left eye. The right eye did not show any acute changes. Findings within the anterior segment of both eyes were unremarkable. 

Investigations

**Figure 2:**
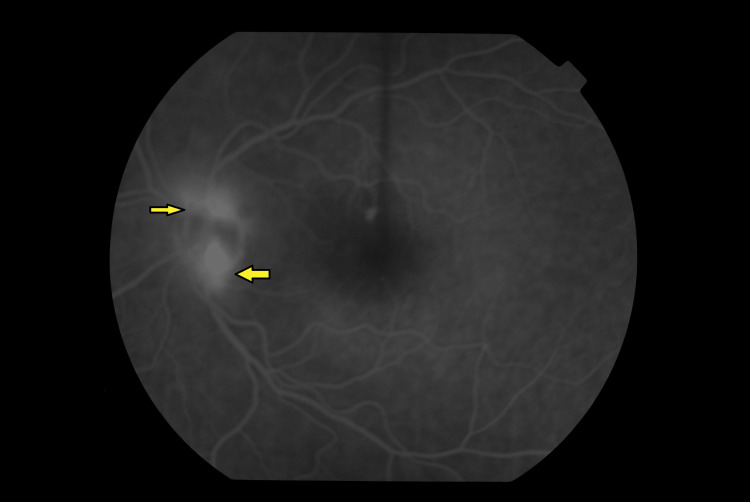
Fundus fluorescein angiography (FFA) image of the left eye. Left optic disc hyper-fluorescence (arrows).

The patient underwent fundus fluorescein angiography that showed hyper-fluorescence on the left optic disc with no areas of capillary dropout (Figure 2). Accordingly, an urgent MRI of both orbits was arranged for the exclusion of compressive left optic neuropathy. The MRI, however, did not show any significant findings. Due to the suspicion of ocular ischemic syndrome, she had carotid Doppler to rule out carotid artery occlusion.

**Figure 3:**
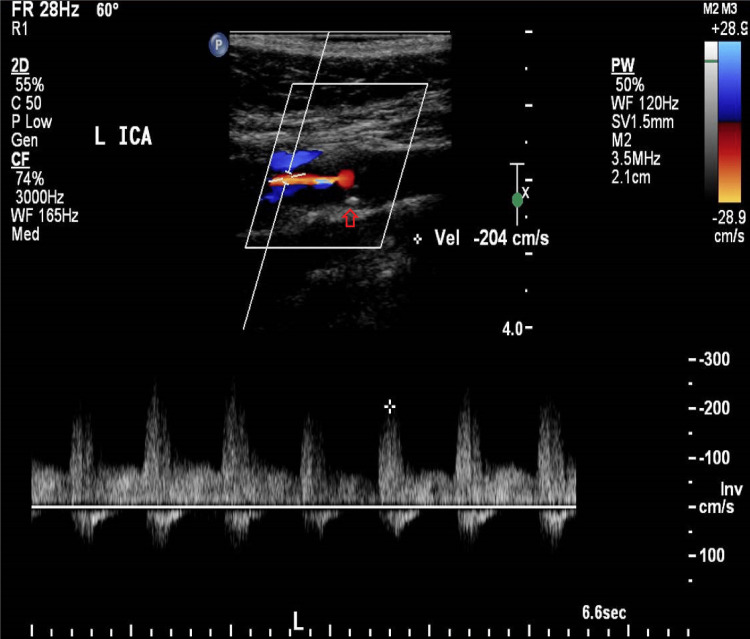
US Doppler of the left carotid. Extensive non-calcified cholesterol plaque (arrow) extending into the left internal carotid artery (LICA) with a peak systolic velocity of 200 cm/s indicating 60-79% stenosis of LICA. US: ultrasonography.

**Figure 4:**
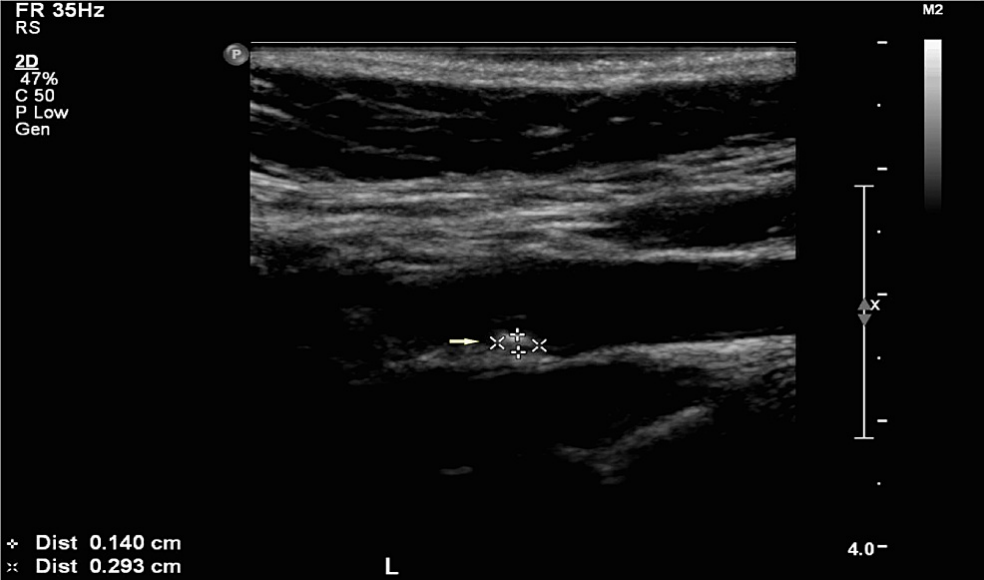
US Doppler of the left carotid. Extensive soft plaque (arrow) at the left carotid bifurcation extending into the left internal carotid artery. US: ultrasonography.

Carotid Doppler demonstrated significant occlusion of 60-79% along the most proximal 2 cm of the left internal carotid artery (ICA) with atherosclerotic plaque extending to the distal part of the artery (Figures 3, 4). Full visualization of the distal portion of the internal carotid artery was not possible. The left external carotid artery (ECA) was patent. The right ICA was patent, but the right ECA showed stenosis of 40-59%.

**Figure 5:**
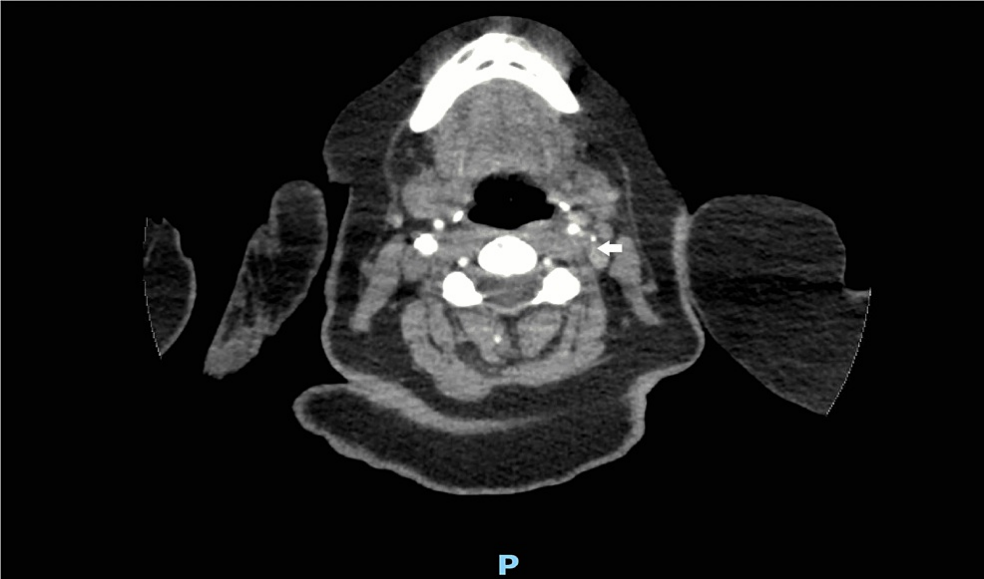
CT angiogram aortic arch and both carotids. Focal critical stenosis within the proximal left ICA (arrow). ICA: internal carotid artery.

The changes in the left ICA were considered a high-priority finding, and therefore, the radiologist referred the patient for an urgent CT angiogram of the aortic arch and the carotids. The CT angiogram scan confirmed critical stenosis in the proximal ICA (Figure 5).

Treatment

As a result of the CT angiogram and the carotid Doppler findings, the patient was referred to vascular surgeons. She underwent a repeat carotid duplex, which showed a progression of stenosis in the left ICA to 80-90%. Weighing the benefits of reducing the risk of future strokes against the risks of the surgery, the patient agreed to and had a carotid endarterectomy.

Outcome and follow-up

Following carotid endarterectomy, the patient had a postoperative follow-up with the vascular team, who were satisfied with the patency of the left ICA. They advised her to continue with statins and aspirin as long-term management.

**Figure 6:**
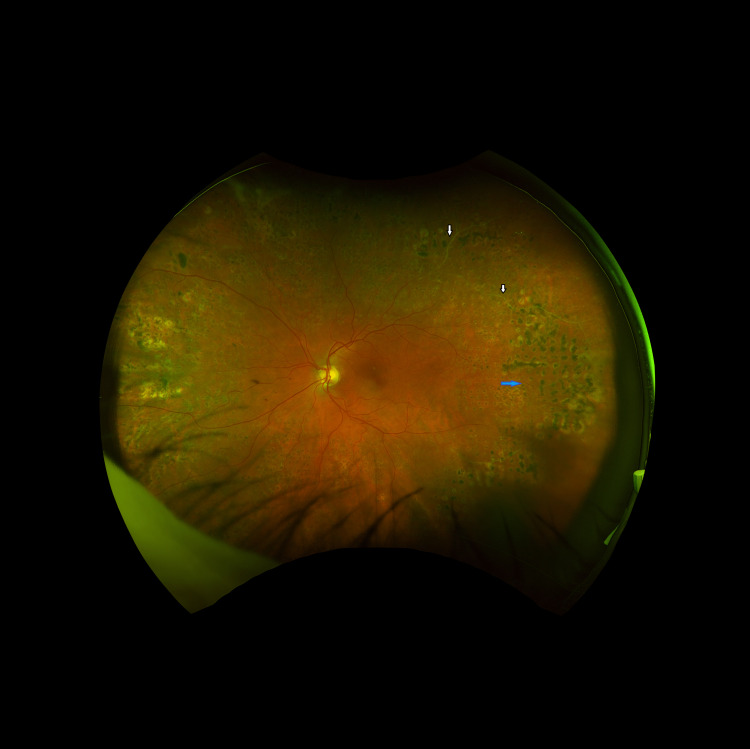
Ultra-widefield retinal image (Optos) of the left eye. PRP scars in the retinal periphery (blue arrow). Occluded vessels were identified previously on the first presentation in the left temporal retina (white arrows). Left NVD stable. PRP: pan-retinal photocoagulation, NVD: neovascularization.

The patient also had regular 6- to 12-month follow-ups in the retinal clinics. Assessment for any possible visual complications due to ocular ischemic syndrome response to the treatment was done throughout. During these follow-ups, she had signs of rubeosis iridis in the left eye. IOP measurements were 19 and 21 mmHg in the left and right eyes, respectively. Accordingly, she had pan-retinal photocoagulation (PRP), which helped in settling ocular changes (Figure 6). Optic disc neovascularization in the left eye remained stable and less active without any further progression. The right eye examination remained unremarkable with visual acuity of 6/6 in both eyes and normal intraocular pressure.

## Discussion

What is ocular ischemic syndrome? What is the pathogenesis? 

Ocular ischemic syndrome (OIS) is a severe form of chronic ischemia caused by chronic hypoperfusion of the anterior and posterior segments of the eye due to ipsilateral carotid artery stenosis [2,5]. The ophthalmic artery, as the first intradural branch of the internal carotid artery, provides a good indication of the vessel's perfusion status [3].

Atherosclerosis is one of the major causes of carotid artery occlusion, and hence the ocular ischemic syndrome [3]. This is reflected in the gender predilection of OIS, with men being twice as likely as women to be affected due to the higher incidence of atherosclerosis in men [1,3,5,6]. 

**Figure 7:**
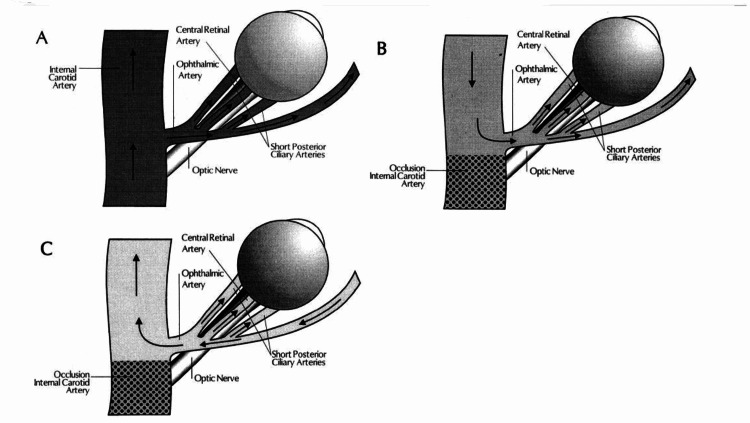
Blood flow in the ophthalmic artery (OA) and its branches. (A) in a normal individual; (B) in a patient with internal carotid artery occlusion and collateral circulation via the circle of Willis (antegrade ophthalmic artery flow); and (C) in a patient with internal carotid artery occlusion and collateral circulation via the ophthalmic artery (reversed flow), and causing reduced blood flow to the eye [1,7].

It has been described that the circle of Willis can provide a collateral blood supply in vascular occlusive events. This pathway acts as a compensatory mechanism for blood flow to the brain, and the blood flow in the ipsilateral ophthalmic artery is antegrade [7]. Reversal of the blood flow in the ophthalmic artery, however, occurs when the anterior and posterior communicating arteries are not capable of maintaining sufficient cerebral blood flow. Thus, establishing communication between internal and external carotid artery branches. This is known as the steal phenomenon [1,7]. This decrease in retrobulbar blood flow increases the risk of the eye developing ischemic ocular changes (Figure 7).

It is also thought that the transition from hypotensive retinopathy to OIS is mediated by the angiogenic factors released from the retina and their spread toward the anterior segment causing iris neovascularization and secondary glaucoma [8].

What are the clinical findings?

Patients’ presentations can vary. Some can be asymptomatic, presenting with incidental ocular abnormalities, and some can have constitutional symptoms [1,5]. As mentioned in this report, our patient did not have any ocular or even constitutional symptoms on referral. The most common manifestation of OIS is vision loss, which can be gradual (80% of the time) or sudden (12% of the cases). Another type of visual loss presentation is amaurosis fugax. Ocular or periorbital pain is another symptom seen in up to 40% of patients [3,6]. Pain can be due to either raised IOP or maybe ischemic in origin. “Ocular angina” has been described in some conditions where the pain is relieved by rest or lying down [1,3,6].

OIS can be seen on examination as changes in both the anterior and posterior ocular segments. Findings are typically unilateral, corresponding to the carotid occlusion side [8]. Rubeosis iridis (67%) is the most common anterior segment change observed in OIS, eventually predisposing to neovascular glaucoma. The most common posterior segment changes are venous stasis retinopathy, which includes narrowed retinal arteries (90%), dilated non-tortuous retinal veins (90%), retina hemorrhages (80%), and microaneurysms (80%) [3,4].

Common causes of optic disc neovascularization (NVD) presentation are usually diabetic retinopathy and retinal vein occlusion. Brown et al. reported NVD due to OIS in 35% of the cases [3,6]. He described the NVD ranged from relatively mild to a fibrovascular stalk extending into the vitreous cavity [6]. Jacobs and Ridgway reported that all six patients presented who had NVD presented with amaurosis fugax as the most common ocular symptom [4]. However, in both pieces of literature, the patients had additional findings ranging from rubeotic glaucoma, episcleral injection or venous stasis, alongside ocular or carotid occlusion symptoms [4,6]. In our patient, however, NVD was an incidental and the sole ocular finding in the left eye with no other symptoms or signs on presentation.

One of the main differential diagnoses for OIS is diabetic retinopathy. In diabetic retinopathy, there is low retinal artery pressure with bilateral and symmetric involvement throughout the fundus compared to retinopathy from carotid artery occlusion. Retinopathy from carotid artery occlusion is usually a unilateral finding with decreased retinal artery pressure in reversed ophthalmic artery blood flow [7,8].

Since most of these patients had atherosclerosis, other co-morbidities were identified, e.g., hypertension 73% and diabetes mellitus 56% [3]. The long-term prognosis is poor. Within five years of onset, the mortality rate in OIS patients has been reported to be as high as 40% due to cardiac problems in most cases (67%) [1,2,3,9]. 

How to diagnose OIS? 

Due to the seriousness of this condition, as has been shown above, prompt early diagnosis and management are required. The American Academy of Ophthalmology guidelines state that ophthalmic or retinal artery occlusion within any age group should prompt the clinician to begin a systematic review for possible thromboembolic disease or carotid occlusion, alongside a vasculitis or hypercoagulability workup if needed. In some cases, they may require urgent referral to a stroke center or neurologist [10]. 

After fundus examination, fluorescein fundus angiography (FFA) is an integral part of investigating OIS [2,3,10]. Delayed and patchy choroidal filling (60%) is observed with a delay of more than one minute, sometimes until complete choroidal filling [3]. It also shows late arterial staining (85%) as a result of ischemia-induced endothelial damage. Other FFA findings are leakage from new vessels or areas of retinal capillary of non-perfusion [3,6]. As has been described above, the FFA in our case showed hyper-fluorescence of the left disc with no areas of capillary dropout. Due to risks of neovascular glaucoma occurrence and noted raised IOP in 50% of OIS cases, the IOP of the affected eye should be monitored [1,9].

In an electroretinogram (ERG), OIS patients show a reduction in a and b wave amplitude, which represent the outer and inner retina layers, respectively. This occurs due to disturbed circulation in the retinal layers [1,3,6]. Other ocular investigations that can be of use include color fundus photography, optical fundus tomography (OCT), indocyanine green angiography, and ocular ultrasonography [10].

In addition to carotid Doppler, MR and CT angiography have shown high sensitivity and specificity in carotid artery occlusion with identification of the degree of stenosis [3]. This was noted in the previously described CT angiography results for our case.

How to manage OIS?

Since OIS is an ocular manifestation of arterial disease, its management is multifaceted, and a multidisciplinary team approach is essential. Because atherosclerosis is the most common cause of OIS, lifestyle modification should be implemented as part of management. Management of associated cardiovascular risk factors and co-morbidities with antiplatelet and statin therapies is essential [10].

Specialist ophthalmic management is directed toward the observed manifestations of the condition. This includes pharmacological treatment of high IOP. In cases of posterior segment neovascularization, PRP is usually recommended. However, PRP has produced guaranteed results as well in cases of anterior segment neovascularization [10]. In 36% of the cases that had pan-retinal photocoagulation (PRP), iris neovascularization showed regression. Sometimes, surgical management is used in chronic irreversible conditions [2,3]. The off-label use of intravitreal anti-vascular endothelial growth factor (VEGF) agents can be helpful to optimize visualization [10]. As shown above, during follow-up, our patient received PRP after the development of rubeosis iridis, which settled her condition.

Vascular team opinion is essential for the management of the underlying carotid artery disease. In our case, that took the form of carotid artery endarterectomy (CEA). When compared to aspirin-only therapy, combined CEA and aspirin therapy reduces the two-year stroke risk rate from 26% to 9% [3]. A better outcome is achieved with CEA management in symptomatic carotid disease with more than 70% carotid artery occlusion compared to medical therapy. Patients should also be started on antiplatelet and statin therapies [10]. Follow-up via both the patient’s physician and ophthalmologist should be arranged as part of monitoring response to treatment and further counseling to avoid recurrence [10].

## Conclusions

Ocular ischemic syndrome, though a rare condition, can lead to serious ocular complications and indicates potential life-threatening conditions. Optic disc neovascularization is one of the rare findings in OIS, which can be an isolated finding. However, findings of ocular neovascularisation should raise strong suspicions of an ischemic cause.

Because of the wide variability of ocular presentations in OIS, high-risk group patients should be followed up frequently to avoid the risks of possible irreversible complications. Upon OIS diagnosis, liaisons with appropriate specialties should be involved to ensure that these patients receive prompt treatment in order to improve their quality of life and avoid potential complications.
